# Leadership and Emotional Intelligence: Current Trends in Public Health Professionals Training

**DOI:** 10.3389/fpubh.2019.00413

**Published:** 2020-01-31

**Authors:** Vladimir A. Reshetnikov, Nadezhda D. Tvorogova, Ilya I. Hersonskiy, Nikita A. Sokolov, Alexander D. Petrunin, Dmitriy A. Drobyshev

**Affiliations:** ^1^Institute of Public Health, Sechenov University, Moscow, Russia; ^2^Institute of Psychological and Social Work, Sechenov University, Moscow, Russia

**Keywords:** emotional intelligence, leadership, public health, professionals training, health-care managers

## Abstract

**Aim of the Study:** The objective of this study was to identify and compare the most significant personal features, and social and psychophysiological characteristics of future health-care managers in order to improve the training program for specialists in the field of health care and public health on the basis of modern concepts about the necessary leadership skills and competence.

**Significance:** The results of the study make it possible to evaluate the effectiveness of career guidance and selection for training future health-care managers on the basis of the application of the following methods: assessing emotional intelligence and taking into account the psychological characteristics of students.

**Materials and Methods:** A total of 242 students of medical and preventive care, residents, and undergraduate students who were trained in the program “Factory of Health Leaders” were included in the study evaluating the level of professional training and indicators of emotional intelligence. Participants underwent testing using a methodology of the management style assessment, as well as testing of their psychophysiological and emotional characteristics.

**Results:** Students who studied in the “Factory of Health Leaders” program had better indicators of emotional intelligence than had ordinary students, but their rates are comparable with residents' rates. This makes us understand that the additional training of emotional intelligence in the process of studying under the higher education program can be successful along with great practical experience in health care as part of residents' curriculum.

**Conclusion:** The training of leadership qualities and emotional intelligence in students by using the example of comparing students in the “Factory of Health Leaders” program with students who have not undergone such training has confirmed its high efficiency and significance in the training of highly qualified personnel in health care.

## Introduction

According to a number of authoritative researchers such as Daniel Goleman, “all effective leaders are united by one essential feature—a high level of development of emotional intelligence” (1). Various studies in this area show us that emotional intelligence (EI) is indeed an indispensable condition for competent leadership, despite first-class professional training, a sharp analytical mind, and various creative personal abilities (2).

The most important among the qualities that a competent manager must necessarily have is EI, the study of which has been paid special attention to in the framework of this study.

As our studies have shown, unfortunately, nowadays, the motivation of students to obtain a specialty in health-care management is often relatively low, and in most cases, it is associated not with the presence of professionally significant qualities but with disappointment in other medical specialties (3).

In this regard, N. A. Semashko, Chair of Public Health and Health Care, has developed a competence-based program of vocational training for students at the stages of higher and additional medical education to prepare competitive specialists in the organization of health care and public health. Within the framework of the activities of Factory of Health Leaders (HLF) student skill laboratory, a series of activities are carried out for their vocational orientation and targeted training, which includes selecting competent students who will comprise a training group, practical orientation of training, and the use of active teaching methods, with motivation of students as the end result.

In addition to educational activities, the main goal of this project is to assess the leadership qualities and the level of EI of future health-care managers.

Currently, there are several interpretations of the term “emotional intelligence.” Psychologists Peter Salovey and John Mayer stated that EI is a section of social intelligence, and they defined EI as the ability to perceive and express emotions, assimilate emotions and thoughts, understand and explain emotions, and regulate emotions (one's own and those of other people) (4). Later, continuing to consider EI as a section of social intelligence, they defined it as the ability to control one's own feelings and emotions and the feelings and emotions of others, to distinguish them, and to use this information to control thinking and actions (5). According to these authors, describing a variety of discrete emotional abilities, EI consists of two parts: emotions and intelligence (emotions relate to a person's feelings in relationships; intelligence refers to the ability to build inferences about something or about someone). EI also consists of four adjacent areas (they are called “branches” to show their hierarchy):

1. identifying emotions (the ability to recognize one's own emotions and the emotions of others);
2. using emotions in solving problems (the ability to evoke emotions and then to use them in thinking);
3. understanding and analyzing emotions (the ability to understand complex emotions and sequences of emotions, and the ability to transfer one emotion to another); and
4. conscious control of emotions (the ability to control both one's own emotions and the emotions of others).

EI, like other types of intelligence, meets three empirical criteria:

1. mental tasks have the right and wrong solutions;
2. measurements of individual mental skills correlate with each other but remain independent measurements; and
3. the absolute level of intellectual abilities increases with age.

There are other models of EI. Thus, American psychologist Daniel Goleman defined EI as the fundamental ability of self-perception, which is expressed in attributes such as respect for others, attentiveness to them, and compassion (Goethe called this property “education of the heart” or cordiality) (6). EI describes a number of human abilities (subtlety of feelings, which is its characteristic feature that contributes to tact, tolerance, “humanity,” etc.) that become character traits. The author believes that the concept of “character” includes the skills that are part of EI. His “mixed model” of EI includes five broad areas:

1. knowledge of emotions;
2. control of emotions;
3. motivation (includes the following skills: the use of emotions to achieve goals, the delayed manifestation of joy and the suppression of impulsivity, and the ability to be in the “general flow”);
4. recognition of the emotions of others; and
5. managing relationships with others.

EI contributes about 80% to personal success [intelligence quotient (IQ)—about 20%]; the level of EI will depend on how a person can treat oneself and others, which will predetermine his/her personal and professional success (6). According to other authors, EI contributes to personal success by about 80% (IQ—about 20%) (6). Success in this case refers to success in a person's relations with others, family, etc.; and the definition of success adopted in society is usually associated with influence, high income, etc. (7).

Today, the development of leadership ability models is very relevant in the preparation of leaders in the health-care system. The main objective of such training is to prepare a competent manager who is able to effectively manage a medical organization staff, possesses a full set of managerial competencies, and most importantly has a systemic vision of global trends in the industry along with risk management skills at both the local and global levels.

According to recognized researchers such as Daniel Goleman, “the difference between an outstanding manager and a good one lies not in education or technical skills, but in the level of development of emotional intelligence, which is represented by a combination of five skills that allow managers to achieve the most effective work not only from themselves, but from their subordinates as well.” His research showed that “when the top management of a company has the necessary level of emotional intelligence, the annual performance indicators of all the divisions is 20% higher than planned” (8). Therefore, for the development and improvement of models of effective management activities, it is necessary to pay special attention to the study of the level of EI of future leaders in the field of health management at an early stage of vocational training.

D. Goleman identifies the following components of EI:

1. self-awareness—the ability to understand one's strengths and weaknesses, values, and motives;
2. self-control—the ability to control or direct the destructive impulses and emotions to a fruitful channel;
3. motivation—the desire to work for the process itself;
4. empathy—the ability to understand the emotional state of other people; and
5. social skills—the ability to build and manage relationships with other people (9).

All the components of EI listed by D. Golemen are personal qualities that we think a manager, a leader in public health, should have.

V.G. Krysko also identified a number of personality traits of managers associated with the level of EI, which can be extrapolated in the development of the health-care system (10).

The ability to focus on the task and people is one of the main characteristics in the profession of a doctor, who is initially focused on humans; one can expect the following strategies of a doctor in a management position:

1. preservation of a humanistic orientation;
2. a shift from the humanistic focus to a focus on the labor performance of workers; and
3. an attempt to preserve both types of behavior.

If task-oriented behavior affects the labor performance of workers more, then the people-oriented behavior is more reflected in the satisfaction of subordinates with their work. The best option would be to use a flexible management style, depending on which result is more important, provided that the task is fulfilled satisfactorily.

Therefore, speaking of the ability to focus on the task and people, we can say that this characteristic is directly related to a component of EI such as emotional awareness and recognition of other people's emotions.

Exceptional activity and consistent activity are the characteristics that should be basic characterological features, manifested in social perseverance, persistence, courage, and endurance. In this case, speaking of exceptional activity and consistent activity, it should be noted that the necessary component of EI for the above characteristics will be the management of one's emotions, because in the absence of this skill, as well as in conditions of emotional stress, active consistent activity is not possible.

A leader in the field of health-care management having a high level of EI is able to most effectively manage a medical organization through the use of effective motivational mechanisms aimed at forming the congruence of the personal tasks of the team and the medical organization itself. He/she shall also be able to respond to the global challenges of the industry, including the most urgent needs of people, dictated by their social and demographic positions, as well as value orientations.

## Materials and Methods

This study was carried out in accordance with the recommendations of Local Ethics Committee of I. M. Sechenov First Moscow State Medical University (Sechenov University). The protocol was approved by the Local Ethics Committee of I. M. Sechenov First Moscow State Medical University (Sechenov University) consisting of the following:

Chairman of the Committee: D. A. Balalykin

Deputy Chairman of the Committee: E. L. Rebrova

Members of the committee: I. I. Ermolaeva, N. G. Berdnikova, N. I. Borisova, E. A. Volkova, V. A. Grigoryan, E.V. Dubogay, and O. A. Subbotina

All subjects gave written informed consent in accordance with the Declaration of Helsinki, Federal Law “On Personal Data” on July 27, 2006 (no. 152-FZ), and the Constitution of the Russian Federation.

In 2016–2018, a study of 242 people was conducted consisting of students, residents, interns, and undergraduates; the students are “oriented” to training in public health and health care profile.

The following are the inclusion criteria of the study:

1. men and women aged 18–60 years;
2. students studying in the “medical and preventive care” program;
3. students studying in “Public Health and Health Care” program and residents of “Organization of Health and Public Health” program;
4. students of the HLF program; and
5. students who gave written informed consent to participate in the study.

Each student had the right to withdraw from the study at any stage.

The comparison group, in this case, was a group of students in residency programs, because these students had experience in health care and because the results of this group could be compared with the indicators of EI and psychophysiological parameters of trained and untrained students.

The main group (72 people) consisted of listeners of the HLF program. The rest of the respondents were assigned to a group of students (104 people) enrolled in the medical and preventive care program and the “residents/undergraduates” group (66 people). A comparative assessment of social and demographic characteristics, level of professional training, and indicators of EI in groups of students was carried out.

At the same time, age, quality of life, marital status, participation in public events, engagement in scientific work, sports, and participation in social activities were studied using the questionnaire survey.

The psychophysiological characteristics of the respondents were also studied using the management style assessment methodology developed by P. Hersey and K. H. Blanchard; the levels of personal and reactive anxiety were also assessed using Spielberger's test; a “well-being–activity–mood” test was conducted; and EI was studied using the technique of N. Hall (11, 12).

The effectiveness of the training of future health managers was analyzed on the basis of the results of the online testing conducted in 2018 within the framework of the Management and Leadership in Health Care Moscow Olympiad.

In our opinion, the technique developed by Nicholas Hall is the most effective tool for studying EI in future health leaders (12). The technique consists of 30 statements divided into five scales: 1) emotional awareness, 2) management of one's emotions, 3) self-motivation, 4) empathy, and 5) recognition of other people's emotions. The answer to each statement implies a 6-point rating (from −3 “completely disagree” to +3 “fully agree”). After the questionnaire survey, with the use of the methods of a statistical analysis, an analysis and a comparison of the characteristics of respondents from different groups were conducted.

## Results

**Figure 1:**
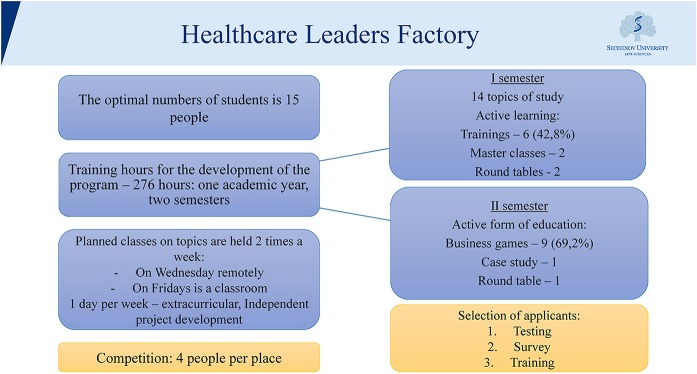
Healthcare Leaders Factory Educational Program.

As it was mentioned above, the main group of subjects for the study of the level of EI in future health managers is a group of students of the educational project developed by the employees of N. A. Semashko Chair of Public Health and Health Care—the Factory of Health Leaders. The structure of the course implemented in this project is shown in Figure 1.

In the course of the training, students of the educational program develop leadership skills, allowing the formation of a pool of potential health leaders and the formation of professional orientation of those who wish to continue training in residency for subsequent appointment to senior positions in the field of health. At the same time, the students improve their communication skills and skills on self-governance. It is important to note that in the course of training in the framework of this program, we use various forms of training: classes at the chair department and in the center; training; small group teaching; and business games. All these forms of training are combined with physical activity.

### Social Portrait and Leadership Characteristics of Students

**Figure 2:**
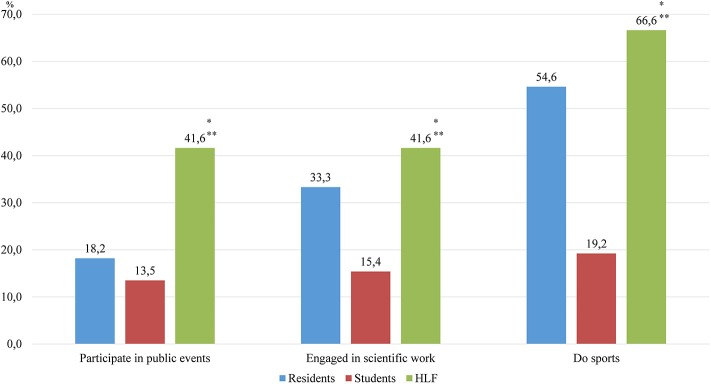
Social activity. *Statistical significate relative to the group of students (*p* < 0.05), **statistical significate relative to the group of residents (*p* < 0.05).

The analysis showed that the majority of respondents who were trained under the HLF program have characteristics such as positive attitudes and values and an attentiveness attitude toward their health (two thirds of the respondents were doing sports). More than half of the respondents from this group participated in the scientific work and public life of the educational institution, as presented in Figure 2. Also it should be noted that one third of the respondents were married.

**Figure 3:**
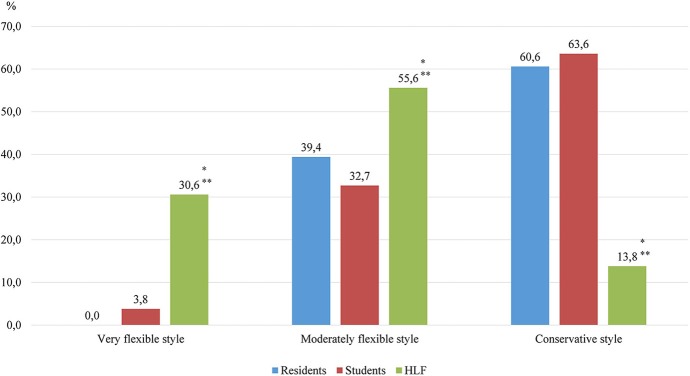
Evaluation of leadership style. *Statistical significate relative to the group of students (*p* < 0.05), **statistical significate relative to the group of residents (*p* < 0.05).

At the next stage of the study, a comparative assessment of the leadership qualities of the participants in the study of different groups was conducted using the assessment of leadership style. As it can be seen in Figure 3, in the group of students under the HLF program, the flexible leadership style was more often characteristic; in particular, more than half of them had a moderately flexible style. At the same time, they had a conservative leadership style that is four to five times less often.

The comparative analysis showed that students have a lower level of personal and reactive anxiety.

The assessment of professional skills was based on the results of the online testing conducted in 2018 within the framework of the Management and Leadership in Health Care Moscow Olympiad. These results indicated that the proportion of correct answers among people who studied under the HLF program was higher compared with the indicators of the other groups; thereby, the level of theoretical knowledge in this group exceeded the corresponding figures in the comparison groups.

Similarly, those who studied under the HLF program had higher levels of assessment of their solution to situational problems, as well as practical skills.

### Characteristics of Respondents' Emotional Intelligence

The HLF students had high levels of empathy and self-motivation. It is known that self-motivation is primarily associated with the life priorities, convictions, and aspirations of a particular person. Self-motivation is one of the main qualities of an effective personality, in both professional and personal aspects of his/her development, which is subject to self-realization. We have found that the high rates of self-motivation suggest that respondents have the ability to pursue the realization of their life plans, set clear goals for themselves, and achieve them.

Empathy is also an important personal and professional quality of a doctor, because of the ability to empathize and perceive the sensory states of another, as well as the presence of skills to establish adequate and emotionally favorable relationships with patients, the ability to analyze their behavior and the behavior of others, human attitude to the patient, ethics, and deep intuition.

At the same time, the average level of empathy is optimal for this category of respondents, because such people try to control their emotional area and try not to show their true feelings to others, which is important for a leader.

Characteristics such as emotional awareness indicate the extent to which respondents can understand themselves, recognize negative and positive feelings and emotions, and understand not only the cause of their occurrence but also the relationship between their own feelings and actions. Emotional awareness allows us to recognize and control our own emotions, which is essential in the practical activities of doctors and organizers. We noticed that this indicator was higher in HLF students than in other groups.

At a higher level, there was also an indicator such as “managing one's emotions,” which implies emotional intransigence and emotional flexibility, in other words, the arbitrary control of one's own emotions.

The results of studies according to the Recognition of Other People's Emotions scale indicate that respondents with high levels can have an effective impact on the emotional state of other people, which in turn is an important professional quality of an organization's physician. As for the groups of respondents who showed an average level according to this given scale, as a rule, such people can also have an impact on their interlocutor, but it is less effective than that of people with a high level according to this scale.

**Figure 4:**
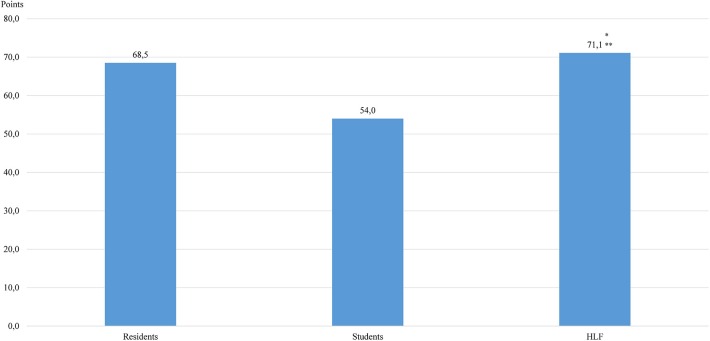
Integral level of emotional intelligence. *Statistical significate relative to the group of students (*p* < 0.05), **statistical significate relative to the group of residents (*p* < 0.05).

As the study showed, the highest integrative level of EI was identified among HLF respondents, the lowest indicators were found among the student groups of respondents, and the average indicators were found among groups of post-graduate students and master's level students, as shown in Figure 4.

## Discussion and Conclusions

The majority of respondents trained under the HLF program have characteristics such as positive attitudes and values and a careful attitude toward their health (two thirds of them were doing sports). More than half of the respondents from this group participated in the scientific work and public life of the educational institution, and one third were married. The comparative assessment showed that these students have characteristics such as higher psychological stability compared with that of other groups, rational strategy of behavior in conflict situations, and optimal characteristics of EI for a future leader, which are high levels of emotional awareness and self-motivation, as well as moderate levels of empathy (Table 1).

**Table 1:** Summary chart of levels of social activity, leadership style, and emotional intelligence.

	**Social activity (%)**			**Leadership style (%)**			**Emotional intelligence (points)**
	**Public events**	**Scientific work**	**Do sports**	**Very flexible**	**Moderately flexible**	**Conservative**	
Residents	18.8	33.3	54.6	0.0	39.4	60.6	68.5
Students	13.5	15.4	19.2	3.8	32.7	63.6	54.0
HLF	41.6	41.6	66.6	30.6	55.6	13.8	71.1

The analysis of the future health managers' training efficiency, based on the results of the online testing conducted in 2018 within the framework of the Management and Leadership in Health Care Moscow Olympiad, showed that the proportion of correct answers among students in this group was significantly higher (*p* < 0.05) than in other groups of participants of the Olympiad who did not undergo training under this program, such as the residents/undergraduate students group and a group of students enrolled in the medical and preventative care program (13). As it can be seen, the HLF trainees had higher characteristics of recognizing the emotions of other people and the integral level of EI than other groups had.

Thus, it can be concluded that the indicators of EI in the persons enrolled in the HLF program are associated with strong leadership qualities and a high level of psychological stability, which in turn confirm the high efficiency of the developed program of elite training: the persons who have been trained under this program have characteristics such as higher professional knowledge and skills than have those who did not undergo such training, as well as a greater degree of leadership qualities.

## Data Availability Statement

The raw data supporting the conclusions of this article will be made available by the authors, without undue reservation, to any qualified researcher.

## Author Contributions

VR conceived of the presented idea and provided a theoretical basis for the research. NS, AP, and DD conducted surveys, testing of respondents, statistical data processing, and contributed to the interpretation of the results. NT and IH conducted a study of EI and assessed the psychophysiological data of the respondents. VR, NS, AP, DD, NT, and IH contributed to the final version of the manuscript.

### Conflict of Interest

The authors declare that the research was conducted in the absence of any commercial or financial relationships that could be construed as a potential conflict of interest.
